# FimH as a scaffold for regulated molecular recognition

**DOI:** 10.1186/s13036-020-00253-2

**Published:** 2021-01-12

**Authors:** Shivani Gupta Ludwig, Casey L. Kiyohara, Laura A. Carlucci, Dagmara Kisiela, Evgeni V. Sokurenko, Wendy Evelyn Thomas

**Affiliations:** 1 Department of Bioengineering, University of Washington, 3720 15th Ave NE. Foege N430P, Box 355061, Seattle, USA; 2 Department of Microbiology, University of Washington, HSB room J267a, Box 357735, Seattle, WA USA

**Keywords:** Recognition molecules, Allostery, Conformational change, Parasteric, FimH, Regulation

## Abstract

**Background:**

Recognition proteins are critical in many biotechnology applications and would be even more useful if their binding could be regulated. The current gold standard for recognition molecules, antibodies, lacks convenient regulation. Alternative scaffolds can be used to build recognition proteins with new functionalities, including regulated recognition molecules. Here we test the use of the bacterial adhesin FimH as a scaffold for regulated molecular recognition. FimH binds to its native small molecule target mannose in a conformation-dependent manner that can be regulated by two types of noncompetitive regulation: allosteric and parasteric.

**Results:**

We demonstrate that conformational regulation of FimH can be maintained even after reengineering the binding site to recognize the non-mannosylated targets nickel or Penta-His antibody, resulting in an up to 7-fold difference in K_D_ between the two conformations. Moreover, both the allosteric and parasteric regulatory mechanisms native to FimH can be used to regulate binding to its new target. In one mutant, addition of the native ligand mannose parasterically improves the mutant’s affinity for Penta-His 4-fold, even as their epitopes overlap. In another mutant, the allosteric antibody mab21 reduces the mutant’s affinity for Penta-His 7-fold. The advantage of noncompetitive regulation is further illustrated by the ability of this allosteric regulator to induce 98% detachment of Penta-His, even with modest differences in affinity.

**Conclusions:**

This illustrates the potential of FimH, with its deeply studied conformation-dependent binding, as a scaffold for conformationally regulated binding via multiple mechanisms.

**Supplementary Information:**

The online version contains supplementary material available at 10.1186/s13036-020-00253-2.

## Background

The ability to trigger binding and release of targets would be highly useful in many areas of biotechnology. Drug delivery, for example, relies on controlling release of the drug payload at the targeted site. Bioseparations takes advantage of capturing and, after flow-through, eluting a desired molecule to isolate or purify it. In diagnostics, sample preparation also benefits from a “capture and release” mechanism for isolating and concentrating desired biomarkers from a sample. In this way, these and many other applications could be improved by the use of regulated recognition proteins.

Several techniques have been shown to create regulated recognition proteins. In drug delivery, cleavable linkers are often added to binding proteins to release the bound drug [1], but this mechanism is irreversible, giving only one-way control upon cleavage. Alternatively, protein domains may be inserted into existing recognition proteins to regulate the activity of the latter [2]. For example, ligand binding at the inserted domain can regulate the parent protein’s enzymatic activity [3] [4], or capture of its native ligand [5]. A particularly notable example is one where calmodulin, which undergoes a large conformational change upon binding its ligand, is successfully inserted into a variety of single-chain variable fragments (scFvs) to introduce affinity modulation [6]. This method essentially builds allostery into proteins that were not originally allosteric, but this very advantage also results in large multi-domain proteins. A simpler approach may be to modify the epitope of an already well-studied, allosterically regulated protein, resulting in a protein scaffold with regulated binding. Scientists often turn to protein scaffolds when generally addressing recognition protein needs, resulting in successful protein scaffolds such as anticalins [7], affibodies [8], affimers [9], DARPins [10], and over a dozen more [11]. More interesting, however, is the use of scaffolds with innate conformation-dependent binding. This was demonstrated with a repeat-in-toxin domain, whose conformational change to a β-roll secondary structure upon calcium binding was successfully retained to capture and release the non-native target lysozyme instead [12]. Therefore, it is worth exploring whether other well-characterized, conformationally-regulated proteins can be modified to recognize a new target and retain conformational regulation of binding. This would provide an additional tool for generating regulated recognition molecules and build upon our fundamental knowledge of the flexibility of conformational regulation.

**Fig. 1:**
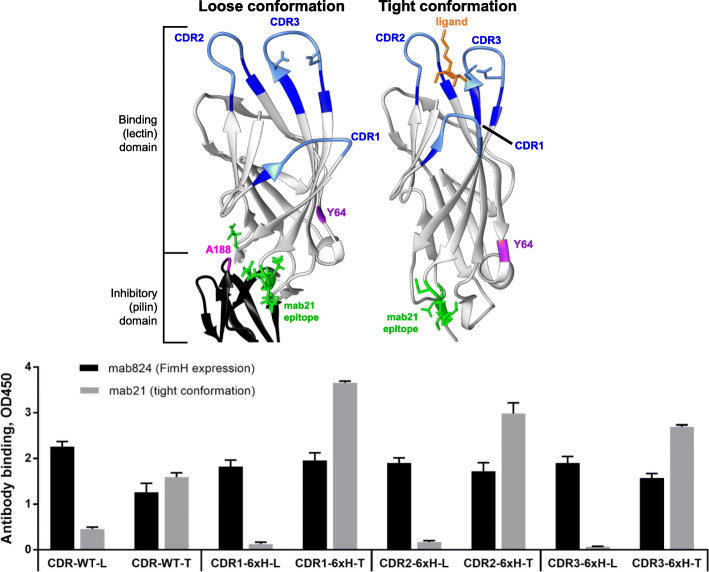
Structure and conformation of FimH lectin domain and the FimH CDR-6xH variants. (a) The crystal structures of the loose (left, PDB ID: 4XO9, [14]) and tight (right, PDB ID: 1UWF, [15]) conformations of FimH are illustrated. Residues of the CDR loops that are mutated to histidine are in light blue (with those critical for mannose binding, N135 and D140, shown with sticks), and the remaining residues of the CDR loops are in dark blue. The rest of the lectin domain is white, the portion of the pilin domain shown is black, the bound ligand heptyl alpha-D-mannopyrannoside is orange, the residues mutated as “tightening substitutions” are pink (A188) and purple (Y64), and lastly, the epitope of mab21 is green (N29, N152-V156) [16]. (b) Binding of pili (CDR-6xH variants or wild-type CDR loops) was tested to mab824 (black bars) for expression and proper folding, and to mab21 (gray bars) to determine expression in the tight conformation. Nonspecific binding was subtracted from all measurements (*n* = 3, mean ± standard deviation)

In this work, we considered the bacterial adhesive protein FimH not only because it undergoes a large conformational change, resulting in significantly different ligand affinities [13], but also because it is conformationally regulated via more than one mechanism, presenting an interesting challenge for creating conformationally-regulated scaffolds. FimH is the last subunit of long organelles called fimbriae, or pili, protruding from *Escherichia coli* cells, and it consists of two domains connected by a flexible linker. The pilin domain, shown in black in Fig. 1a, anchors FimH to the rest of the pili via a donated β-strand, while the lectin domain, shown in white in Fig. 1a, binds to mannose (and, in fact, a wide range of terminal mannosylated compounds, including heptyl alpha-D-mannopyrannoside, shown in orange in Fig. 1a). The mannose-binding pocket on the lectin domain contains three exposed loops that interact with mannose; we refer to these as the “CDR” loops to equate them functionally with antibodies’ complementarity-determining regions. These may be ideal sites for mutation to recognize alternative ligands. The pilin domain allosterically regulates binding of mannose via noncovalent interactions with the lectin domain, causing the mannose-binding pocket of FimH to accept a “loose” conformation that has low mannose affinity [13]. These noncovalent interactions may stochastically break, inducing a conformational change in the binding pocket of FimH to tighten around mannose (“tight” conformation), resulting in a high-affinity, long-lived interaction with mannose [13]. In the absence of any trigger, this happens infrequently in wild-type FimH, which strongly prefers the loose conformation. However, certain mutations or triggers can force FimH to switch to the tight conformation. A well-studied example of this trigger is tensile force from fluid drag during bacterial adhesion in flow, resulting in formation of “catch-bond” type interactions between FimH and its ligand that are strengthened by force [17] [13].

Triggers other than force can also affect the conformational state of FimH. Some monoclonal antibodies (mab) raised against FimH exhibit conformational specificity. One such antibody, mab21, recognizes FimH and allosterically stabilizes its tight conformation. It does so by binding at a site distal from the CDR loops (shown in green in Fig. 1a), where it obstructs the noncovalent interactions between the two domains to enable the higher mannose affinity [16] [18]. This raises the question of whether mab21 can still allosterically regulate binding of FimH after the CDR loops are mutated to recognize different ligands. Another trigger is mannose itself. Since it stabilizes FimH in the tight conformation, it may also promote this state in mutated FimH if its epitope remains intact. This behavior is observed in the presence of the antibody mab926, which binds adjacent to mannose in the binding pocket of FimH, where it stabilizes the loose conformation. Since the epitope of mab926 only partially encompasses the binding site of mannose, mab926 and mannose exhibit “parasteric” behavior, a noncompetitive mechanism of inhibition between two ligands with overlapping binding sites [19]. Importantly, soluble mannose, in the form of methyl α-D-mannopyranoside, has been shown to forcibly dissociate mab926 from FimH, which was possible because mannose could bind its epitope and induce the tight state, distorting the mab926 association with FimH. This suggests that mannose could be used as a parasteric trigger for dissociation of other ligands that bind adjacent to mannose in the pocket but preferentially recognize the loose conformation.

FimH has been altered to recognize new targets previously [20] [21], but those variants were neither designed nor tested for regulated binding, since the regulation of FimH was not understood at the time. We hypothesize that by deliberately mutating only regions of FimH known to contribute to ligand specificity, away from the regulatory region, we can introduce binding to new targets that take advantage of the conformation-dependent regulation of FimH via allosteric and “parasteric” mechanisms.

Here, we show that FimH can serve as a conformationally-regulated scaffold for generating regulated recognition proteins. We demonstrate that all three CDR loops of the FimH pocket are permissive to mutations that can result in recognition of new targets, without inhibiting the conformational changes of FimH. While some of these new protein variants may bind their targets with equal affinity in both conformations, we identify several variants that have conformation-dependent binding to their new target using either parasteric or allosteric effectors.

## Results

### The CDR loops in the FimH binding pocket carry positions that are permissive to substitution in both conformations

To test whether the CDR loops of FimH carry positions that can be varied without destabilizing the protein or preventing conformational changes, a hexa-histidine tag (6xH) was substituted into each loop. This tag was chosen as a model epitope that has been used previously for testing for permissive sites [22]. In the CDR1 loop (^10^AIPIGGG^16^), positions I11 to G16 were replaced with six histidine residues, or 6xH. The same was done to D47 to I52 in the CDR2 loop (^46^NDYPETITD^54^) or to N135 to D140 in the CDR3 loop (^133^QTNNYNSDDF^142^). All mutations were made to the K-12 variant of the *fimH* gene, which we hereafter refer to as CDR-WT-L, because it has wild-type binding loops and prefers the loose conformation in the absence of any regulator [14]. These constructs were made in plasmid pBAD-Fim, which has the full structural *fim* operon downstream of the *araBAD* promoter, enabling expression of FimH at the tips of type 1 fimbriae. We refer to the resulting FimH variants as CDR1-6xH-L, CDR2-6xH-L, and CDR3-6xH-L (Table 1), where the “-L” reminds us that these variants are expected to prefer the loose conformation. For expression of pili containing these FimH variants, the plasmids were introduced into *E. coli* MegaX DH10B™ cells, a *fim* null strain.

**Table 1:** Effect of conformation on affinity of FimH for Penta-His

FimH Variant	Mutation(s)	Penta-His K_D_ (nM) w/o effector	Penta-His K_D_ (nM) with effector	Observed regulation by effectors	Effect of conformation on binding to Penta-His
CDR1-6XH-L	11–16➔6xH	170 ± 16^a^	N/A		Higher affinity in tight conformation
		114 ± 9^b^	28 ± 3 man^b^	*activated*	
CDR1-6XH-T	11–16➔6xH A188D	38 ± 4^a^	32 ± 3 mab21^a^	*no effect*	
		34 ± 3^b^	13 ± 1 man^b^	*activated*	
CDR2-6xH-L	47–53➔6xH	7.5 ± 0.4^a^	N/A		Higher affinity in loose conformation
		N/A	N/A		
CDR2-6xH-T	47–53➔6xH A188D/Y64R	21 ± 1^a^	51 ± 3 mab21^a^	*inhibited*	
		40 ± 9^b^	120 ± 20 man^b^	*inhibited*	
CDR3-6xH-L	135–140➔6xH	2.8 ± 0.3^a^	N/A		No effect of conformation on affinity
		N/A	N/A		
CDR3-6xH-T	135–140➔6xH A188D/Y64R	3.6 ± 0.2^a^	3.7 ± 0.2 mab21^a^	*no effect*	
		N/A	N/A		

^a^ Affinity for Penta-His determined in Fig. 3 (with or without mab21)

^b^ Affinity for Penta-His determined in Fig. 5 (with or without man (mannose))

N/A indicates that this was not applicable because the CDR-6xH variant did not bind the effector in question (mab21 binding determined in Fig. 1 and mannose-binding determined in Fig. 4)

Expression of properly-folded FimH was confirmed by binding of the anti-FimH monoclonal antibody 824 (mab824) to surface-immobilized pili in an ELISA. Mab824 is suitable for this because it recognizes both loose and tight conformations equally (Fig. S1), at a tertiary epitope distal from the CDR loops [19]. As shown in Fig. 1b, all four “L” variants were recognized by mab824 at similar levels, showing that mutation of the CDR loops did not affect FimH expression. The anti-FimH monoclonal antibody 21 (mab21) was used to determine the conformation of each variant because it only recognizes FimH in the tight conformation [16]. As shown in Fig. 1b, all four “L” FimH variants were not recognized by mab21, confirming that all preferred the loose conformation. Indeed, the introduction of 6xH into any of the three loops decreased mab21 binding over that observed for CDR-WT-L, suggesting that the bulky histidine residues favor the loose conformation of the pocket.

Therefore, in order to engineer FimH in the tight conformation with these same 6xH-modified loops, additional substitutions, A188D and Y64R, were introduced. We refer to these as “tightening substitutions” because they have been shown to stabilize the tight conformation of the lectin domain [23, 24]. They also lie in regulatory regions far from the CDR loops, as shown in Fig. 1a. We refer to the resulting variants as CDR-WT-T (wild-type loops with A188D), CDR1-6xH-T (CDR1-6xH with A188D), CDR2-6xH-T (CDR2-6xH with A188D and Y64R), and CDR3-6xH-T (CDR3-6xH with A188D and Y64R), where the “-T” reminds us that these variants prefer the tight conformation. This is confirmed via mab21 binding as shown in Fig. 1b, where the addition of A188D was sufficient to enable maximal mab21 binding for FimH with wild-type and CDR1-6xH loops, but FimH with CDR2-6xH and CDR3-6xH additionally required Y64R to induce maximal mab21 binding.

Together, these studies demonstrate that while replacements to the CDR loops may preferentially stabilize one conformation of FimH, it is possible to express FimH with replaced CDR loops in both conformations by introducing additional substitutions into the regulatory region.

### Substitutions in the FimH CDR loops introduce binding to a new target for probing conformational regulation

**Fig. 2:**
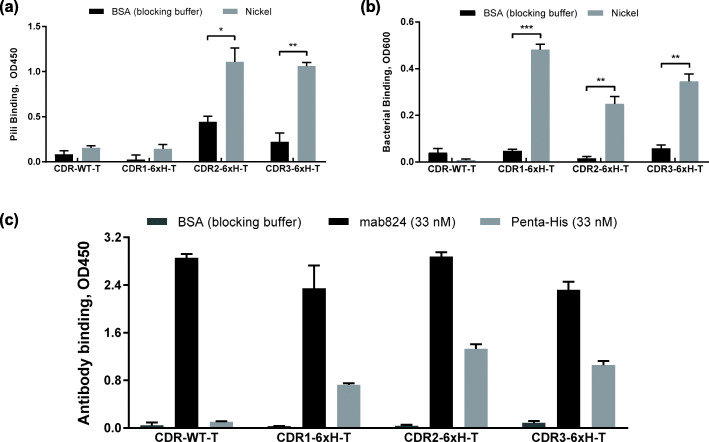
Binding of CDR-6xH variants to nickel and to Penta-His antibody. (a) Pili in the tight conformation (CDR-6xH variants or wild-type CDR loops) were bound to nickel-coated plates (gray bars) or uncoated plates (black bars) blocked with BSA, then detected using mab824 (*n* = 3, mean ± standard deviation, *** *p* ≤ 0.0005, ** *p* ≤ 0.005, and * *p* ≤ 0.05). (b) *E. coli* cells expressing CDR-6xH variants were added to nickel-coated plates (gray bars) or to uncoated plates (black bars) blocked with BSA (*n* = 3, mean ± standard deviation, *** p ≤ 0.0005, ** p ≤ 0.005, and * p ≤ 0.05). (c) Pili in the tight conformation (CDR-6xH variants or wild-type CDR loops) were immobilized and incubated with BSA (dark gray bars), mab824 as a control (black bars) or Penta-His antibody (light gray bars) (*n* = 3, mean ± standard deviation)

The use of 6xH as a model epitope allows for probing conformational regulation against a non-mannosylated, non-native ligand of FimH. Therefore, we first tested whether substitution of 6xH was successful in enabling FimH to recognize a new target via the binding pocket. The variants CDR1-6xH-T, CDR2-6xH-T, and CDR3-6xH-T were tested for binding to nickel by incubating their pili with Ni (2+) chelate-coated wells (hereinafter, the Ni (2+) chelate is referred to simply as nickel) and detected with mab824 in an ELISA. Figure 2a shows that variants CDR2-6xH-T and CDR3-6xH-T, but not CDR1-6xH-T, bound well to nickel at a pili concentration of 8.9 nM. As a more sensitive test of binding, adhesion of whole *E. coli*, which express hundreds of fimbriae, was measured to nickel-coated wells in comparison with control microtiter wells without nickel. Figure 2b shows that all three CDR-6xH variants tested, including CDR1-6xH-T, successfully bound nickel. Similarly, we then tested these three CDR-6xH variants for binding to the anti-6xH antibody Penta-His. Figure 2c shows that 33 nM Penta-His recognized immobilized pili expressing each of the three CDR-6xH variants, but not CDR-WT-T. We therefore have two new targets, nickel and Penta-His, against which we can now probe whether conformational regulation was retained.

### CDR-6xH variants have conformation-dependent affinity for non-mannosylated targets

**Fig. 3:**
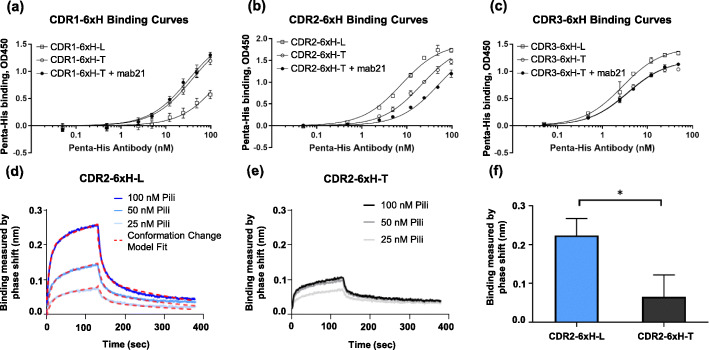
Conformational dependence of binding by CDR-6xH variants to Penta-his antibody. (a) Binding of Penta-His to CDR1-6xH variants using ELISA, where K_D_ = 169 ± 16 nM (loose conformation, open squares), 38 ± 4 nM (tight conformation, open circles), 32 ± 3 nM (tight conformation + mab21, filled circles). (b) Binding of Penta-His to CDR2-6xH variants using ELISA, where K_D_ = 7.5 ± 0.4 nM (loose conformation, open squares), 21 ± 1 nM (tight conformation, open circles), 52 ± 3 nM (tight conformation + mab21, filled circles). (c) Binding of Penta-His to CDR3-6xH variants using ELISA, where K_D_ = 2.8 ± 0.3 nM (loose conformation, open squares), 3.6 ± 0.2 nM (tight conformation, open circles), 3.70 ± 0.20 nM (tight conformation + mab21, filled circles). (d) Sample data for binding of CDR2-6xH-L pili to Penta-His using bio-layer interferometry (BLI), with a fit to a two-state (conformation change) reaction model. (e) Sample data for binding of CDR2-6xH-T pili to Penta-His using BLI showing that binding is not concentration-dependent. (f) Binding measured at the end of the association phase during BLI, just before the dissociation phase, for both variants of pili. Binding curves using ELISA (*n* = 3, mean ± standard deviation) were fitted using GraphPad Prism 7 software using a 1:1 binding model. Error bars in BLI graphs represent the standard deviation of *n* = 2 experiments on separate days. * indicates *p* < 0.05 by 1-tailed Student’s t-test

The affinity of the CDR-6xH variants in each conformation for Penta-His was measured in an ELISA by varying the concentration of the antibody and fitting the results with a 1:1 binding model. For each modified CDR loop, we tested the affinity of the mutant: 1) on its own, 2) upon introducing tightening substitutions, and 3) with tightening substitutions and upon treatment with mab21. The affinity of the CDR1-6xH variants for Penta-His was higher in the tight conformation, because the K_D_ decreased 5-fold from 169 ± 16 nM to 38 ± 4 nM with the tightening substitutions, with or without mab21 (Fig. 3a). In contrast, the affinity of the CDR2-6xH variants for Penta-His was higher in the loose conformation, because the K_D_ increased by 3-fold, from 7.5 ± 0.4 nM to 21.4 ± 1.1 nM, with the tightening substitutions in CDR2-6xH-T. Adding mab21 to CDR2-6xH-T further increased the K_D_ over 2-fold, to 51 ± 3 nM (for a total of 7-fold increase over CDR2-6xH-L), demonstrating mab21’s potential to act as an effector (Fig. 3b). Lastly, the affinity of the CDR3-6xH variants for Penta-His was similar in both conformations (Fig. 3c), because the K_D_ was 2.8 ± 0.3 nM vs. 3.6 ± 0.2 nM with and without the tightening substitutions, and adding mab21 did not change the K_D_. These observations are summarized in Table 1.

In order to confirm the conformation-dependence of affinity of the CDR-6xH mutants, we measured binding of pili to immobilized Penta-His using Bio-Layer Interferometry (BLI), which offers label-free, real-time kinetics and affinity data. The CDR2-6xH variants were chosen as the test case for this method, to confirm that tightening substitutions led to a difference in affinity. In the shorter timescale of these experiments, the binding of CDR2-6xH-L to Penta-His was relatively complex, involving both fast and slow dissociation kinetics. This could not be fit with a 1:1 binding model, but fit a two-state reaction binding model well (Fig. 3d). Importantly, wild-type FimH similarly shows two-state binding to mannose, in which the two states differ much more in kinetics than in affinity [25]. In this two-state fit, the measured K_D_ of CDR2-6xH-L to Penta-His was 62.5 ± 33.3 nM (*n*=3). In contrast, CDR2-6xH-T demonstrated low Penta-His binding (Fig. 3e), where the level of binding was near the observed noise level for BLI (0.1 nm) [26], even at concentrations 2 to 5 times higher than the K_D_ of CDR2-6xH-T to Penta-His as measured using ELISA. While it is possible that the low binding level could be quantified with either higher pili concentrations or longer association times, these modifications would make this technique unaffordable, so neither BLI nor other real-time binding techniques were further pursued for affinity data. Nevertheless, because the response after 120 s of association with 100 nM of CDR2-6xH-L pili is significantly higher than that with CDR2-6xH-T pili (Fig. 3f), it is clear that the CDR2-6xH variants bind better to Penta-His in the loose than in the tight conformation in this assay as well, validating the conformation-dependent binding observed via ELISA. Therefore, we continue to use ELISA for remaining affinity measurements.

### The affinity of CDR-6xH variants for Penta-his can be affected by mannose

**Fig. 4:**
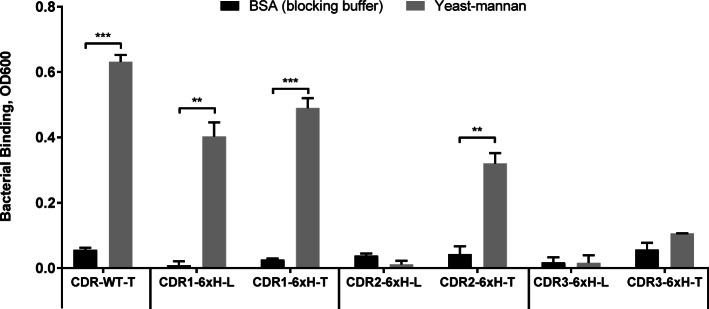
Bacterial adhesion assay showing binding by cells expressing CDR-6xH variants to mannose. Cells expressing each variant were bound to yeast-mannan to measure mannose binding (gray bars) or to uncoated plates (black bars) blocked with BSA (*n* = 3, mean ± standard deviation, *** p ≤ 0.0005, ** *p* ≤ 0.005, and * *p* ≤ 0.05)

It was previously demonstrated that by inducing the tight conformation, mannose can displace the pocket-targeting parasteric antibody mab926. This suggests that mannose might regulate conformation-dependent binding of FimH to new targets, but only if the FimH mutants retain the ability to bind mannose. We therefore first tested if any of the CDR-6xH variants retained binding to mannose. A bacterial adhesion assay was performed in which bacterial cells were incubated with surfaces coated with the mannosylated substrate, yeast-mannan, used in many mannose-binding studies for FimH [16, 21, 23, 24]. Figure 4 shows that the CDR1-6xH variants bound mannose in both conformations. This is interesting because it suggests that even though CDR1-6xH-L prefers the loose conformation (Fig. 1b), it can still bind mannose, potentially by shifting to the tight conformation, although at a slightly lower level than CDR1-6xH-T. On the other hand, CDR2-6xH-T bound mannose, but CDR2-6xH-L did not, suggesting that in this case, the 6xH substitution in CDR2 more strongly stabilized the loose conformation of FimH, preventing mannose from forming long-lived interactions. The CDR3-6xH variants did not bind mannose well even in the tight conformation, suggesting that key mannose-binding residues were replaced by this substitution.

**Fig. 5:**
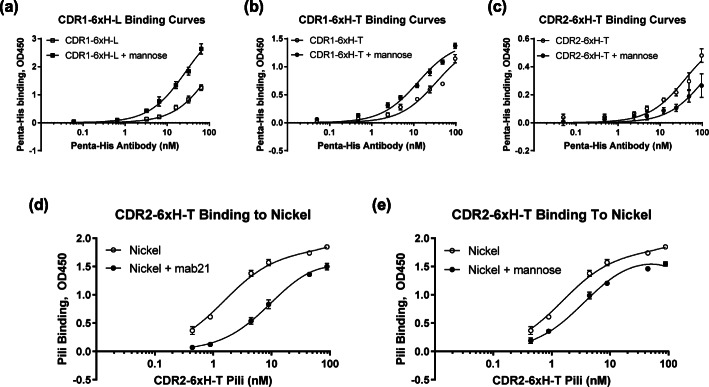
Effect of mannose or mab21 on CDR-6xH variants’ binding to Penta-his antibody or nickel. (a) For CDR1-6xH-L to ligand Penta-His antibody: K_D_ = 114.20 ± 9.26 nM without mannose (open squares) and 27.70 ± 2.84 nM with mannose (filled squares). (b) For CDR1-6xH-T to Penta-His: K_D_ = 34.54 ± 3.47 nM without mannose (open circles) and 12.71 ± 1.46 nM with mannose (filled circles). (c) For CDR2-6xH-T to Penta-His: K_D_ = 39.61 ± 8.93 nM without mannose (open circles) and 116.1 ± 21.5 nM with mannose (filled circles). (d) For CDR2-6xH-T binding to nickel, K_D_ = 1.53 ± 0.12 nM without mab21 (open circles) and 10.24 ± 0.70 nM with mab21 (filled circles). (e) For CDR2-6xH-T to nickel: K_D_ = 1.53 ± 0.12 nM without mannose (open circles) and 3.72 ± 0.27 nM with mannose (filled circles). Binding curves (*n* = 3, mean ± standard deviation) were fitted using GraphPad Prism 7 software using a 1:1 binding model

For the CDR-6xH variants that strongly bound mannose, we next tested whether soluble mannose (2% methyl α-D-mannopyranoside) affects their affinities for Penta-His. The K_D_ of CDR1-6xH-L for Penta-His decreased 4-fold from 114 ± 9 nM to 28 ± 3 nM when mannose was added (Fig. 5a), while the K_D_ of CDR1-6xH-T decreased 3-fold from 34 ± 3 nM to 13 ± 1 nM with mannose (Fig. 5b). These observations are consistent with our above findings that the CDR1-6xH variants bound Penta-His best in the tight conformation, which can be induced or further stabilized with the addition of mannose. In contrast, the K_D_ of CDR2-6xH-T for Penta-His increased 3-fold from 40 ± 9 nM to 116 ± 22 nM when mannose was added (Fig. 5c), consistent with our above findings that the CDR2-6xH variants bound Penta-His best in the loose conformation. These observations are also summarized in Table 1.

In summary, we observed different patterns of conformational regulation for the CDR-6xH variants. The CDR3-6xH variants are not conformation-sensitive and do not bind mannose well, so are of limited interest for studying regulation of binding to the new targets. The CDR1-6xH variants can be induced by mannose to bind Penta-His more strongly. Most interesting, though, is the CDR2-6xH-T variant, which binds Penta-His and nickel best in the loose conformation. We showed that its binding to Penta-His is regulated by both effectors, mannose (Figs. 5c) and mab21 (Figs. 3b). This form of regulation is inhibition, which opens the possibility of using the effectors to reverse binding. Therefore, for the remainder of this study, we explore the CDR2-6xH variants in greater detail.

### The affinity of CDR2-6xH variants for nickel can be regulated by conformation

Here we asked whether either mab21 or mannose could also regulate binding of CDR2-6xH-T to nickel. First, pili ranging from 0.44 to 89 nM were incubated with Ni (2+) chelate-coated wells with and without mab21, which would further stabilize the tight conformation (Fig. 5d). The K_D_ of CDR2-6xH-T for nickel was increased nearly 7-fold by mab21, from 1.5 ± 0.1 nM to 10.2 ± 0.7 nM. Similarly, the K_D_ of soluble CDR2-6xH-T pili binding to nickel plates was increased 3-fold by mannose’s ability to further stabilize the tight conformation, from 1.53 ± 0.12 nM to 3.72 ± 0.27 nM (Fig. 5e). These findings demonstrate that binding to nickel can also be inhibited by both soluble effectors.

### Mannose and mab21 induce detachment of CDR2-6xH-T from both targets

**Fig. 6:**
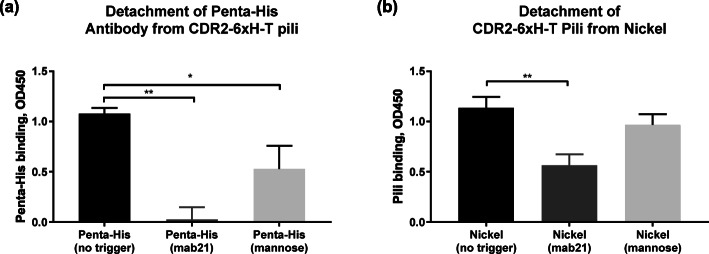
Detachment of ligand from CDR2-6xH-T in ELISA. Bound ligand (Penta-His antibody or nickel) is detected after one hour of dissociation in buffer alone with no trigger (black bars), in buffer with mab21 (dark gray bars), or in buffer with mannose (light gray bars). Nonspecific binding was subtracted from all measurements (*n* = 3, mean ± standard deviation, ** p ≤ 0.005, and * p ≤ 0.05)

Since the affinity of CDR2-6xH-T for both Penta-His and nickel is reduced by the addition of mannose or mab21, we next asked if CDR2-6xH-T could be detached from Penta-His or nickel using mab21 (10 μg/mL fab fragments) or free mannose (2% methyl α-D-mannopyranoside), even with modest changes in K_D_. In these detachment tests, the same ELISAs were used as described previously, except that, after binding of Penta-His to immobilized pili or binding of soluble pili to immobilized nickel, the wells were incubated for 1 h with either just buffer, mab21 fab fragments, or free mannose. The ‘no trigger’ control condition measures Penta-His that remains after any dissociation that occurs in buffer alone, without a trigger. Any dissociation beyond this is therefore induced by the trigger. After one hour, mab21 and mannose each induced detachment of Penta-His from CDR2-6xH-T pili by 98% ± 11% (*p* = 0.0011) and 51% ± 22% (*p* = 0.0471), respectively (Fig. 6a). For nickel, only mab21 induced significant detachment of pili, by 50% ± 13% (*p* = 0.0031) (Fig. 6b). Thus, for CDR2-6xH-T, mab21 can allosterically induce detachment of both Penta-His and nickel, and mannose can parasterically induce detachment of Penta-His, demonstrating regulatory behavior by two effectors with different mechanisms.

## Discussion

Here, we describe a new method for generating regulated recognition proteins to a target, where regulation can include both capture and release of the target. We demonstrate that the allosteric bacterial protein FimH can be used as a conformation-dependent scaffold for creating regulated binding proteins. Beyond its regulatory mechanisms, FimH has many characteristics that are desired for a scaffold for protein engineering, such as: 1) a well-defined hydrophobic core for a stable framework [10], 2) a solvent-accessible pocket, and 3) a relatively small size. While FimH is naturally a two-domain protein of 34 kDa, several point mutations have been shown to eliminate the requirement of the regulatory domain so that the 17 kDa binding domain alone reproduces the allosteric function [27]. That is, one can create even smaller allosteric regulatory proteins from FimH. Overall, this approach to engineering regulated recognition proteins may have advantages over other methods, including retaining the protein’s smaller size and reversible regulation.

We first show that the CDR loops of FimH carry positions permissive to substitution. Because we are using FimH for its conformation-dependent binding, our definition of ‘permissive positions’ includes those which, after mutation, allow for not only FimH expression, but also for assuming both loose and tight conformations. This was shown with the 6xH tag. While histidine residues in the loops appeared to cause all CDR-6xH variants to favor the loose conformation, possibly due to the histidine residues’ bulk, we show that both point mutations and, in the case of CDR1-6xH, mannose, can be used to shift the conformational equilibrium as needed. Thus, although mutations in the loop regions may bias the conformation of FimH, they do not preclude the use of FimH as a regulatable scaffold.

We also demonstrate that changes to the CDR loops of FimH result in binding to new, non-mannosylated targets, allowing us to probe whether conformational regulation is retained against these new targets. Most display scaffolds bind only to large molecules such as proteins; binding to small molecules is notoriously difficult [28]. In fact, one of the few non-immunoglobulin scaffolds to bind small molecules is anticalin, based on the lipocalin fold. This scaffold and FimH both have a deep binding pocket capable of capturing small molecules. Since FimH’s natural target is the small molecule mannose, we expect FimH would be an ideal scaffold for a range of targets that included large proteins and small molecules. For this study, we use the model epitope 6xH to conveniently probe conformational regulation against nickel and Penta-His antibody, yet it would be worthwhile to explore the potential diversity of new ligands that can take advantage of FimH’s conformational regulation.

The ability of some of the variants with modified CDR loops to retain binding to mannose is significant, because mannose controls the conformation of FimH and is a small, inexpensive soluble molecule that can be washed away as well as added, allowing rapidly reversible regulation. It is therefore worth analyzing which residues can be substituted without losing the ability to bind mannose. Mannose touches over a dozen FimH residues in crystal structures [29], but molecular dynamics simulations [30] and mutagenesis studies [31] show that only residues F1, N46, D54, Q133, N135, and D140 are critical for mannose binding. In contrast, residues I13, D47, Y48, I52, F142 form part of the binding pocket according to crystal structures [29], but do not appear to be critical for binding to mannose. While all the CDR substitutions we performed included residues in the binding pocket, none of the critical residues were modified in CDR1-6xH (residues 11 to 16) or CDR2-6xH (residues 47 to 53), explaining why these variants still recognized mannose. In contrast, two critical residues (N135 and D140, shown in light blue with sticks in Fig. 1a) were modified in CDR3-6xH (residues 135 to 140), explaining why this variant had weaker mannose binding. This suggests that mannose binding at the base of the loops would likely be maintained as long as F1, N46, D54, Q133, N135, and D140 are conserved, especially if only the tops of the loops (residues 11 to 16, 47 to 53, and 136 to 139) were substituted in a diversity library or an engineered variant. That is, the CDR loops are permissive not just in terms of allowing expression and conformational regulation, but also in maintaining mannose binding, if the substituted regions are carefully selected.

In investigating conformation-dependent affinity in the CDR-6xH variants, it was interesting that each loop behaved differently. CDR3-6xH showed little conformation-dependence in its affinity for Penta-His, despite a large movement of loop CDR3 between the two states in wild-type FimH [13]. CDR1-6xH, however, showed an increase in affinity for Penta-His when shifted to the tight conformation, and CDR2-6xH showed a *decrease* in affinity for both Penta-His and nickel when shifted to the tight state. Since different conformation-dependent binding behavior is observed for the same 6xH substitution, tightening substitutions, and effectors, these loops thus provide an experimental control for each other, demonstrating that conformation indeed, not the effectors or tightening substitutions in themselves, influenced Penta-His binding by altering the Penta-His binding sites in the CDR loops. These effects on affinity were highly reproducible. Tightening mutations had the same effect on binding of the CDR2-6xH variants in both ELISA and BLI assays. Moreover, three independent ways of controlling conformation (mutations, mannose, and an antibody) all had the expected effect on affinity for each CDR. The largest difference in K_D_ measured was a 7-fold change, between CDR2-6xH-L and CDR2-6xH-T+mab21 for Penta-His, and between CDR2-6xH-T and CDR2-6xH-T+mab21 for nickel. This change in K_D_ was much smaller than the effect of conformational regulation on the K_D_ of FimH for various mannosylated compounds, which can be up to 500-fold [17, 23]. This may reflect the greater conformation dependence of three-dimensional versus linear epitopes. It is possible that greater conformation dependence may be achieved by simultaneously modifying two or three of the loops shown here to be permissive.

Even with a relatively small change in affinity, though, the CDR2-6xH variants provide the opportunity to test for triggered dissociation. Unlike orthosteric inhibitors, both allosteric [32] and parasteric [19] inhibitors can enhance unbinding rates, and therefore might induce dissociation of ligand. This raises the question of whether mab21 and mannose, which stabilize the tight conformation, are noncompetitive inhibitors that induce dissociation of the CDR2-6xH variants from Penta-His and nickel, which they recognize in the loose conformation. Mab21 binds distal from the 6xH substitution, so is clearly an allosteric noncompetitive inhibitor. The mannose binding site overlaps slightly with the 6xH substitution, so mannose could in theory be either a competitive inhibitor with mutually exclusive binding, or a parasteric inhibitor with overlapping binding sites that can be filled simultaneously due to flexibility in the pocket. Both mab21 and mannose can induce detachment of Penta-His or nickel from CDR2-6xH-T, confirming that both can exert noncompetitive inhibition. Mab21 was particularly effective, inducing nearly 100% dissociation of Penta-His. The effectiveness of the inhibitor in inducing dissociation is far beyond what might be predicted from its effect on apparent affinity, which may reflect a much greater effect on binding kinetics than on affinity, as seen with the parent FimH [25]. This is especially notable given the high affinity which both Penta-His and nickel have for CDR2-6xH-T via the 6xH-tag. Thus, for applications such as drug delivery and purification that would benefit from triggered detachment, our data suggest that large effects on affinity may not be necessary to achieve near complete dissociation of the target. This result therefore demonstrates that conformational regulation can be highly effective in controlling the binding of a FimH mutant to a new target.

## Conclusions

Ultimately, we show that both parasteric and allosteric regulatory mechanisms of FimH can be retained after modifying the binding pocket of FimH. This has not been done previously with FimH, despite its well-characterized conformational behavior. While the affinity changes described have been small, larger affinity changes could be made possible through directed evolution using assays that select for increased affinity in only one conformation. Even with small affinity changes, we have shown that up to 98% detachment can be induced in some cases, which highlights how FimH can be studied for potential use in various “capture and release” biotechnology applications. Thus, FimH as a conformationally-regulated scaffold opens many future possibilities towards generating regulated binding molecules.

## Methods

### Construction of pBAD-Fim mutants

Strain pBAD-Fim was constructed using *Escherichia coli* cells from the MegaX DH10B™ T1^R^ Electrocomp™ cell line (Invitrogen™, Carlsbad, CA), a high-transforming derivative of the *fim* null *E. coli* K12 strain [33]. The strain was transformed with the recombinant plasmid pBAD-Fim (10 kb), made from pBAD/HisB (Invitrogen). This contains the full *fim* operon (excluding regulatory subunits *fimB* and *fimE*), inserted into the vector just downstream of its *araBAD* promoter using Gibson Assembly [34]. Point mutations, substitutions, and insertions were introduced to the *fimH* gene in pBAD-Fim using the QuikChange II XL Site-Directed Mutagenesis kit (Agilent Technologies, Santa Clara, CA, USA). Primers were designed using the QuikChange Primer Design tool (Agilent Technologies). Mutations were verified by sequencing.

### Anti-FimH antibodies

Mouse anti-FimH monoclonal antibodies mab21 and mab824 were described previously [16, 19]. Fab fragments of mab21 were generated using Pierce™ Fab Preparation Kit (Thermo Fisher Scientific, Waltham, MA).

### Pili purification

Fimbriae were purified from the indicated *E. coli* pBAD-Fim strains. Bacteria were grown overnight at 37 °C in LB with 100 μg/mL ampicillin, sub-cultured at a 1/100 dilution for three hours, and fimbrial expression was induced with 0.2% w/v arabinose overnight. Cells were harvested, and pili purified as described previously [16]. Protein concentration was measured using the Pierce™ BCA protein assay kit (Thermo Fisher Scientific, Waltham, MA) after the pili were heated for 5 min at 99 °C in 0.1 M HCl.

### Bacterial adhesion assays

Bacterial adhesion to yeast-mannan was measured using a crystal violet-based assay as described previously [19]. Adhesion to nickel was measured using Pierce™ nickel coated clear 96-well plates (Thermo Fisher Scientific). *E. coli* pBAD-Fim strains were grown as described above for pili purification except that induction with arabinose was maintained for 3–4 h, and 2% methyl α-D-mannopyranoside (Sigma-Aldrich) was added during induction to prevent aggregation observed with high expression levels of wild-type FimH, presumably due to interaction with a carbohydrate on *E. coli*. Harvested and washed cells were added to the plates at OD_600_ of 4.0 for adhesion assays.

### Elisa

Microtiter plates were coated with purified pili at 0.1 mg/mL in 0.02 M NaHCO_3_ buffer for 1 h at 37 °C, washed, and blocked for 30 min with 0.2% BSA/PBS. Primary antibody was added in blocking buffer at concentrations ranging from 0.01 μg/mL to 20 μg/mL for the anti-6xHis antibody Penta-His (Qiagen, Hilden, Germany), or 5 μg/mL for mab824 or mab21. When noted, 2% methyl α-D-mannopyranoside or 10 μg/mL mab21 fab fragments was added with the primary antibodies. After incubation for one hour at 37 °C and 3 washes, HRP-labelled goat anti-mouse IgG Fc secondary antibody (Thermo Fisher Scientific, Waltham, MA) was added at 1:2000 dilution. Following washes, 3,3′,5,5′-tetramethylbenzidine, or TMB (KPL, Gaithersburg, MD), was added, the reaction stopped with 0.3 M sulfuric acid, and absorbance measured at 450 nm.

A different ELISA was performed to test binding of pili to nickel-coated plates. Pili in blocking buffer were bound to nickel for one hour at 37 °C. After washes with PBS, mab824 was added at 5 μg/mL for 45 min before detection with secondary antibody incubation as described above. Another ELISA was performed in which, after Penta-His antibody or nickel binding, a one-hour detachment period followed with either buffer, 10 μg/mL mab21 fab fragments, or 2% methyl α-D-mannopyranoside. After detachment, pili were washed twice. In the case of nickel binding, mab824 was added as described above. Then, mab824 or Penta-His antibody were detected with the HRP-labelled goat-anti-mouse IgG Fc secondary antibody.

### Bio-layer interferometry

Bio-layer interferometry kinetic assays of purified pili binding directly to Anti-Penta-HIS (HIS1K) Biosensors (ForteBio) was performed in a baseline buffer (BB) of HBS-EP (0.01 M HEPES pH 7.4, 0.15 M NaCl, 3 mM EDTA, 0.005% (v/v) Surfactant P20, pH 7.4) with 0.2% BSA, and 0.1% Tween 20 using an Octet RED96 system (ForteBio). HIS1K sensors were first hydrated in BB for at least 10 min. The sensors were then dipped in BB for a 120-s baseline step before being transferred to wells containing 25–100 nM of purified pili in BB for 125–130 s. After this association step, the sensors were transferred back to the original BB wells for 250–400 s of dissociation time. These data were exported using Octet Software and fit to a two-state (conformational change) reaction binding model using BIAevaluation 2.0.4 software (GE Healthcare).

### Statistical methods

Unless otherwise stated, statistical analyses were performed on GraphPad Prism 7 software (GraphPad, La Jolla, Ca) using either multiple two-tailed t-tests (with corrections for multiple comparisons using the Holm-Šídák method) or two-tailed Welch’s t-tests (to correct for unequal variance) comparing each test condition with the control condition. When BLI was used to confirm the difference already observed in ELISA, a one-tailed t-test was used.

## Supplementary Information

**Additional file 1: Fig. S1.** ELISA of mab824 binding to FimH pili in different conformation. (a) Binding is shown to pili in the loose conformation (dark bars) or the tight conformation (light bars), with or without the free mannose, demonstrating no significant difference in binding between the two conformations. (b) Binding is shown to FimH pili in the tight conformation with or without mab21, demonstrating mab21’s lack of impact on mab824 binding.

## Data Availability

Data sharing is not applicable to this article as no datasets were generated or analyzed during the current study.
