# Supplementary figures and images for: FimH as a scaffold for regulated molecular recognition
Source: J Biol Eng. 2021 Jan 12;15:3. doi: 10.1186/s13036-020-00253-2 (PMC7805223; doi:10.1186/s13036-020-00253-2)

(a)

Binding to mab824 (13 nM)

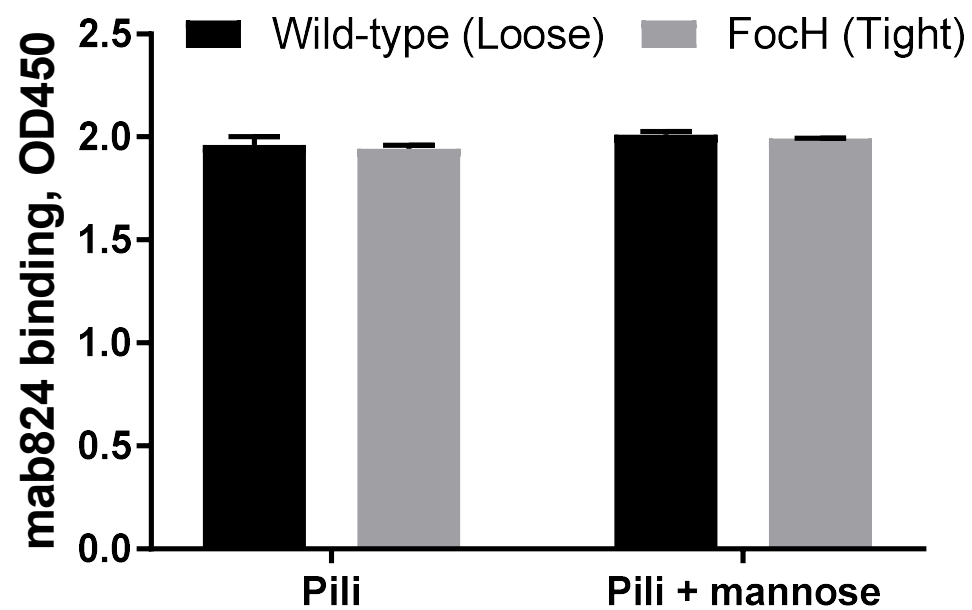

(b)

Binding of FocH Pili to mab824 (13 nM)

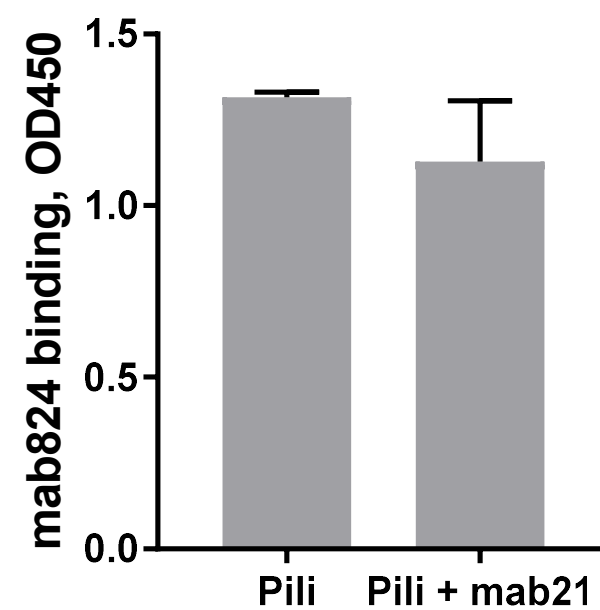

Supplement: Supplementary file 1 — Additional file 1: Fig. S1. ELISA of mab824 binding to FimH pili in different conformation. (a) Binding is shown to pili in the loose conformation (dark bars) or the tight conformation (light bars), with or without the free mannose, demonstrating no significant difference in binding between the two conformations. (b) Binding is shown to FimH pili in the tight conformation with or without mab21, demonstrating mab21’s lack of impact on mab824 binding. [file 13036_2020_253_MOESM1_ESM.pdf]
